# Supplementary material for: Quantitative cell-based model predicts mechanical stress response of growing tumor spheroids over various growth conditions and cell lines
Source: PLoS Comput Biol. 2019 Mar 8;15(3):e1006273. doi: 10.1371/journal.pcbi.1006273 (PMC6538187; doi:10.1371/journal.pcbi.1006273)
Supplement: S1 Text — This text contains more information about the model algorithms and parameters, model calibration and parameter sensitivity. (PDF) [file pcbi.1006273.s001.pdf]

Supplementary information

Below further technical details on the models (Figure A) and parameter sensitivity are given (Figure B). A flow chart of the model algorithms executed in the simulations is depicted in Figure C.

Center-based model: friction terms and forces

We look at the Equation of motion (4) for cells in more detail. The general form for the friction tensors in Equation (4) reads

$$\Gamma_{ij} = \gamma_{\perp}(\vec{u}_{ij} \otimes \vec{u}_{ij}) + \gamma_{||}(I - \vec{u}_{ij} \otimes \vec{u}_{ij}), \tag{0.1}$$

with $\vec{u}_{ij} = (\vec{r}_j - \vec{r}_i)/||\vec{r}_j - \vec{r}_i||$, where $\vec{r}_i$, $\vec{r}_j$ denote the position of the centers of cell i and object j. I is the 3×3 identity matrix, $\otimes$ denotes the dyadic product [12]. The individual cell friction coefficients are $\gamma_{\perp}$ and $\gamma_{||}$, respectively perpendicular and parallel to the movement direction. As experiments do not indicate ECM inhomogeneity or anisotropy, the cell-ECM friction matrix is considered to be diagonal. The ECM was not represented explicitly as experimental observations indicated that the ECM fraction is small and approximately homogeneously and isotropically distributed in the intercellular spaces.

As represented in Equation 5 the interaction force resulting from compression, deformation and adhesion can be expressed as a sum of a repulsion force, here represented by a modified Hertz contact force, and an adhesion force. The interaction force acts along the line connecting the centers of two interacting spherical cells.

The micro-motility force of cell i is mimicked by a Brownian motion term with zero mean value and are uncorrelated in time:

$$< \vec{F}_{mig,i} >= 0 \text{ with } < \vec{F}(t_1)_{mig,i} \otimes \vec{F}(t_2)_{mig,i} >= A\delta(t_1 - t_2). \tag{0.2}$$

Based on a formal analogy to colloidal particle systems [19], the autocorrelation amplitude of the force as approximated $A = 2D\gamma^2 I$, D being the cell diffusion constant, γ the friction coefficient of a cell in the medium and I the unity matrix. The scalar γ emerges in an isotropic environment, for which $\gamma_{||} = \gamma_{\perp}(:= \gamma)$. In an extracellular matrix environment, A is largely controlled by the cell itself [3]. The equations of motion (4) do not conserve total momentum due to the micro-motility term as part of the momentum is transferred to the ECM, that is not explicitly modeled here.

The system of Equation 4 was integrated numerically until the simulation time surpasses the total duration of the experiment which is $\sim 10d$. Equation 4 results in a linear problem with a sparse symmetric positive definite matrix, which can be solved efficiently by a Conjugate Gradient method [12,13] with Jacobi preconditioner to obtain the cell velocities and an explicit Euler integration scheme to obtain the cell postions.

Deformable Cell Model: friction terms and forces

We recapitulate the basic equation of motion for a DCM denoted in (Equation 13). The individual elements generating the in-plane elastic forces between the surface nodes represented by the 1st terms on the lhs and rhs of Equation 13 are modeled by classical linear spring-damper systems. The force between the nodes captures the elastic response of the shell-like structure including the cortical cytoskeleton of the cell. When the elastic and dissipative components are summed up, one acquires a Kelvin-Voigt element. The vector force between two nodes i and j reads (see Figure A):

$$\vec{F}_{ij} = \vec{F}_{e,ij} + \vec{F}_{v,ij} = -k_s(\vec{d}_{ij} - \vec{d}_{0,ij}) - \gamma \vec{v}_{ij}, \tag{0.3}$$

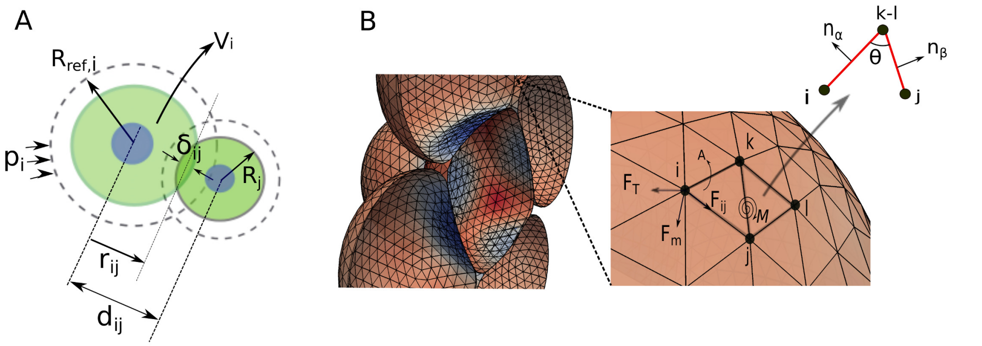

Fig A. (A) Contact between two cells defining (a) the distance d_{ij}, virtual overlap δ_{ij}, reference radius $R_{ref,i}$, actual radius R_i and volume V_i. (B) (left) DCM representation of several adjacent cells. (right) Detail of nodal structure building cell surface and depicting the forces that work on them.

where $\vec{F}_{e,ij}$ and $\vec{F}_{v,ij}$ are the elastic and dissipative forces, k_s is the spring constant, γ represents the dissipation, $\vec{d}_{0,ij}, \vec{d}_{ij}$ are the initial and actual distance vectors, and $\vec{v}_{ij}$ is the relative velocity between the nodes, respectively.

The second term in Equation 13 representing the surface bending resistance is incorporated by the rotational resistance of the hinges determined by two adjacent triangles $\alpha = \{ijk\}$ and $\beta = \{ijl\}$. This defines the bending moment M:

$$M = k_b sin(\theta - \theta_0), \tag{0.4}$$

where k_b is the bending constant, and θ is determined by the normals to the triangles $\vec{n}_\alpha, \vec{n}_\beta$, with θ_0 being the angle of spontaneous curvature. M can be transformed to an equivalent force system $\vec{F}_m$ acting on each of the nodes of the triangles [13]. Restoring volume compression / expansion forces $\vec{F}_{vol,i}$ controlled by the bulk compression modulus of the cytoplasm are computed from the internal pressure using the volume change and bulk modulus of the cytoplasm K. In our simulations K reflects the overall cell compressibility including the cortex, as in its physiological range of elasticity, the cortex contributes little to the overall bulk modulus of the cell, i.e. $K \gg E_{cor} h_{cor} / R_{cell}$ ([8], own test runs). In analogy to Equation 8 the pressure applied to the cell is therefore approximately given by:

$$p = -K \log(\frac{V}{V_0}). \tag{0.5}$$

The forces $\vec{F}_{vol}$ on the nodes are perpendicular to the cell surface and can be obtained by multiplying the pressure with the surface area assigned to each node (each node has 6 adjoining triangles which each count for 1/3 of the surface area).

During the simulations, large variations in area of the triangles in the network can cause numerical artifacts. These can be avoided by adding a force F_T, which is proportional to the area expansion of the individual triangles [15, 20]:

$$F_T = k_A (A - A_0)/A_0. \tag{0.6}$$

Here, A_0 and A are the initial and the current areas of the triangle, and k_A is the area compression stiffness. The forces $\vec{F}_{T,i}$ are summed over all triangles of the cell and transferred to the nodes in the direction perpendicular to the opposite vertex edge of that node leading to increase (or decrease) the area of one triangle at the expense of a decrease (or increase) of its neighbor triangles. We chose k_A such that the influence on

the cortex elasticity is minimal, yet triangle deformations are minimized. Note, these forces do not induce shear elastic effects in the network.

Finally, the interactions between neighboring cells are accounted for by introducing repulsive forces ($\vec{F}_{rep,i}$) and the adhesive forces ($\vec{F}_{adh,i}$) between nodes belonging to different cells. The interaction forces were obtained as detailed in [4, 20]. The model allows simulating the interaction between two arbitrarily shaped triangulated bodies but also between a triangulated body and non-triangulated simple geometric objects as spheres and flat substrates.

Importantly, the parameters of the spring network in Equation 0.3 can be related to macroscopic elastic constants, approximating the cell cortex by a thin shell. For the six fold symmetric triangulated lattice on the cell surface, the linear spring constant k_s can be computed from the Young modulus E_{cor} of the cortex with thickness h_{cor} by [14, 16, 18]:

$$k_s \approx \frac{2}{\sqrt{3}} E_{cor} h_{cor} \tag{0.7}$$

Similarly, the bending stiffness of the cortex can be approximated by

$$k_b \approx \frac{E_{cor} h_{cor}^3}{12(1-\nu^2)}, \tag{0.8}$$

where ν is the Poisson ratio (= 0.3 for an equilateral 2D network of linear springs).

"Global" calibration approach, only valid for confined spheroid

The relationship between forces, inter-cellular volume fraction and pressure established in section "Local" calibration approach, needed for experiment II can also be computed following a simpler, global approach in case of a confined volume as in experiment I. If compression rates are sufficiently slow as is the case in that experiment, the cells in the spheroid can reorganize, distributing the stress isotropically and homogeneously over the cells in the spheroid. For the intercellular spaces in the capsule the *global* intercellular volume fraction ϵ_{int} and the cellular fraction ϵ_{cells} are related by $\epsilon_{int} + \epsilon_{cells} = 1$ with $\epsilon_{cells} = V_{cells}/V_{caps}$. We can now use ϵ_{cells} to re-parameterize Equation 14 assuming cells are homogeneously distributed over the spheroid during compression (compare Figure 8A). The apparent contact stiffness $\tilde{E}_i$ in that case increases for all cells equally with ϵ_{cells} being the equivalent of $1 - \tilde{d}_{ij}$ in the previous approach.

With both different calibration methods, local and global, the CBM simulation results closely follow the DCM curve (Figure 8D). However, the curve using the global calibration method is smoother than for the local calibration method as it represents an average over all cells thus disregarding local fluctuations. For high pressures ($p > 2\,\mathrm{kPa}$), both curves become nearly parallel. On the other hand, as the local approach does not require the existence of an enclosed (capsule) volume it can be used more generally as a cell-cell interaction force upon volumetric compression in many configurations as in experiment II.

We finally point out that no explicit representation of ECM has been considered in the model based on the observations the ECM fraction is rather small (personal communication) and approximately homogeneously and isotropically distributed in the intercellular spaces.

Model parameter and algorithm sensitivity

We also studied the potential influence of parameters calibrated for the free growth conditions which could not be directly inferred from the experiments. For the friction

terms, the cell-cell friction, cell -ECM friction and cell-capsule friction were varied between 1 % ("low") and 200 % ("high") of their reference values. The effect is shown in Figure BA and indicates that even for low friction coefficients the results remain largely unaffected. For the cell-cell adhesion energy W and cell motility coefficients D, which were varied between 10 % and 500 % [6] , we did not observe any significant changes (Figure BE-F). The strength of cell-cell adhesion has been shown to play a role in detachment, but to be of minor importance for multicellular systems under compression (e.g. [11]). Note, that also in MCSs growing in absence of externally applied forces cells are moderately compressed [10].

In conceptual analogy to experimental statistical procedures, we have performed growth expansion simulations in the thin capsule with the optimal parameter set but four different random seeds for the cell-specific cycle time, Young modulus and necrotic pressure threshold to test the effect of stochasticity (see Table 2, Figure BB). Even after more than 7 days of simulation, only very little mutual differences can be observed, while the slopes of the curves are the same. This can be attributed to self-averaging effects such that the variations on the level of individual cells cancel out at the population level (e.g. ref. [7]).

Comparison of calibration methods

For the local calibration procedure, we used the following constants in Equation 14 assuming strain hardening: $a_0 = 0.4454 \cdot 10^5$, $a_1 = 2.347 \cdot 10^5$, $a_2 = -7.918 \cdot 10^6$, $a_3 = 6.615 \cdot 10^8$, $a_4 = -1.206 \cdot 10^9$, $a_5 = -3.091 \cdot 10^9$, $a_6 = 1.1239 \cdot 10^{10}$, yielding a force cell - intercellular distance curve that matches well with the one obtained from the deformable cell simulations experiment (Figure 8). The function for $\tilde{E}(\tilde{d}_{ij})$ is monotonically increasing (Figure BD). For the global calibration approach, we obtained a similar curve. In test simulations for the MCS growth in the thin capsule using contact inhibited growth for both local and global calibration, we found only a mutual variation of $5 - 7\%$, see Figure BC. On the other hand, if no calibration was assumed, a largely unrealistic capsule dilation was obtained (Figure BC), and a consistent relation between pressure and cell density could not be established.

Influence of cell division algorithms

As far as either the volume or the duration of the cell in the cell cycle have passed a threshold value, we replace the mother cell by two daughter cells which are placed very close to each other [17, 22]. This algorithm is different from the approach where cells slowly deform into dumbbells in mitosis phase before splitting into two daughter cells [11, 21]. In our model two daughter cells are created next to each other instantaneously. During the mitosis period (which takes about $1.5h$) the daughter cells do not grow. However, the two newly created cells can generate short-time artificial pressure peaks which disappear during the division time course due to small local re-arrangements. To test the impact of the pressure peak formation on our growth simulation results we implemented an smoothing algorithm that reduces these peaks by ensuring that (1) the mother cell divides in the direction of the least stress as derived from the local stress tensor (see section Measures for stress and pressure), and (2) a local energy minimum is sought by varying the distance between the daughter cells and computing the interactions with the other cells. While the smoothing algorithm reduces the short-time pressure peaks, we did not see significant differences in the results compared to simulations where this algorithm had been dropped.

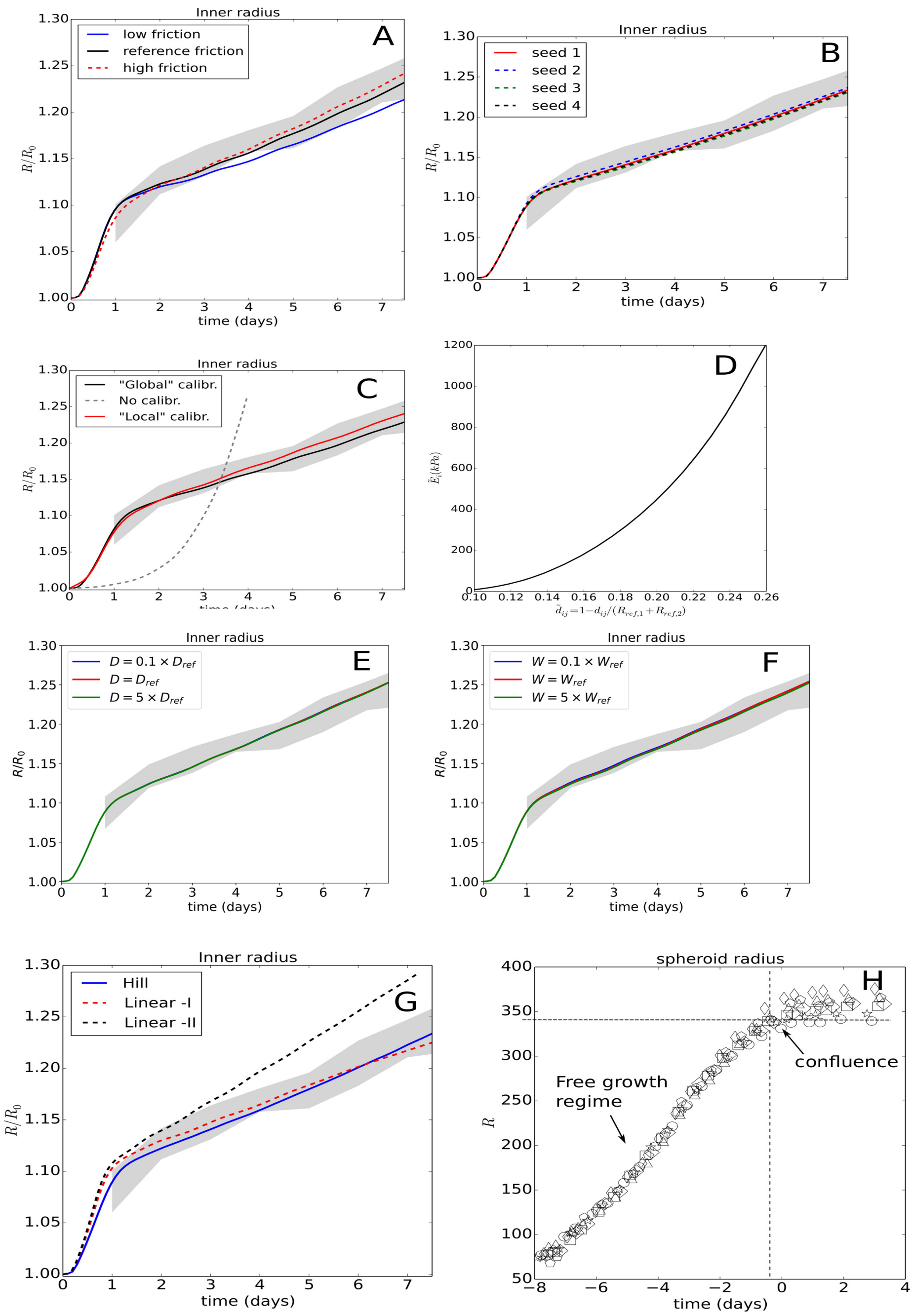

Fig B. (A) Dependence of simulation results of the thin capsule on a variation on the friction coefficients. (B) Comparison of 4 simulations with different random seed. (C) Comparison of the global and local calibration approach for a growing spheroid in the capsule. (D) Plot of the function $\tilde{E}(\tilde{d}_{ij})$ used for cell contact stiffening upon spheroid compression. (E-F) Comparison of 3 simulations of the thin capsule experiment with different cell-cell adhesion energy W, and cell motility D (G) Simulated thin capsule dilatation using for cell cycle progression: the proposed Hill-type function, the linear function (”Linear -I”) as depicted in Figure 3 (which fails to explain the data in experiment II, see Figure 7), and a linear function (”Linear -II”) optimized to match the CT26 spheroid expansion in Experiment II (but here fails to reproduce the thin capsule data). (H) Data plots of MCS growth in a thick large capsule ($\sim 400\,\mu$m) growth. The stress-dominated growth regime is too short to identify the stress-response.

Future extension: possible ways to include extracellular matrix

An important future extension would be the inclusion of extracellular matrix (ECM), which is why we briefly outline how this can be implemented within the current model framework.

There are three approaches with different degree of detail and effort of implementation, one requiring an explicit model of ECM, while two of them might be readily implemented with modest effort.

(i) Approach 1: A sophisticated way would be to model the ECM explicitly e.g., as a network of visco-elastic springs (e.g. Ban et al., 2018 [23]). Such an approach is expensive both implementationwise and in terms of computing power. Alternatively, the effect of ECM can be estimated within two approximations within the framework of the established model.

(ii) Approach 2: One way is to perform the same simulation and calibration but now assuming that there is an ECM substance present in the capsule. To compute the influence of ECM of compression modulus K_{ecm}, one could assume that the ECM is compressed homogeneously and isotropically (shear elastic effects with cells are neglected). In a next step the volume occupied by the (DCM) cells and ECM within the capsule would be estimated prior to compression of the capsule simulation (see Figure 8A) to obtain a corrected relation between cell-cell interaction force and cell-cell distance (Figure 8C) that includes the compression and deformation forces of the ECM. For the Dextran experiment, the volume of the MCS could be estimated by the volume inside an elastic membrane enclosing the MCS and touching its surface.

The pressure necessary for ECM compression could be calculated from Equation 8 whereby dV then denotes the deviation of the ECM volume from its uncompressed volume as a consequence of compression leading to a relation $p(V)$ for the ECM alone. In the compression simulation used to determine the intercellular forces with cell-cell distance, the reduction of cell-cell distance would now cause both a compression of ECM by reducing its volume, and a deformation of the cells. Both, ECM and cell now resist the compression whereas in neglecting ECM, only the resistance of the cells was traced. As the volume of ECM can be calculated at any time from the difference of capsule volume and total volume of all cells, the pressure generated by compression of ECM during the compression simulations can be calculated, and taken into account in the calculation of the force on the cell by adding a force to each node that is proportional to the area to the node's associated cell surface area. This extends the curves in Figure 8C,D by the effect of ECM. For small compression modulus the force would be expected to be small. If at the same time, the compression modulus of the cell would be much larger than order (kPa) (see the discussion of cell compression moduli in the introduction), the repulsive branch of the curves in Figure 8 would become steeper. Approach 2 is a global approximation as it needs information from the entire MCS (the MCS or capsule volume) to estimate the effect of ECM and the simplest approximation.

(iii) Approach 3: The approximation could be based on a composite material approach in which the object that in the main body of the paper mimics the deformable cell, would instead be associated with a tissue unit composed of the cell plus a shell of ECM (for the concept in agent-based models, see Drasdo et. al. (2007) [11]). To determine the relation between force and cell-cell distance in such an approach, the radius of the object now composed of cell and ECM shell would be chosen larger than the cell radius alone to reflect the observed volume percentages of cells and ECM inside the MCS. The Young modulus of the cell cortex in the current approach would be replaced by an effective Young modulus taking into account the Young modulus of the ECM and the Young modulus of the cell cortex. As a simple illustrative example in point mechanics, it is conceptually equivalent to replacing two linear springs of spring constants k_1 (e.g. for the cell cortex) and k_2 (e.g. for the ECM) connected in series by

one effective spring with spring constant $k = 1/(1/k_1 + 1/k_2)$ (e.g. for cell cortex plus ECM). Similarly, the compression modulus of the cortex (that emerges from its Young modulus and Poisson ratio) can be replaced by an effective compression modulus, that other than the spring constants also contain the thicknesses of ECM and the cellular cortex. The effective compression modulus again contains information on compression moduli and volumes. This makes it possible to use the same approach as in the body of this paper by only re-parameterization of the cortex variables and the cell radius. However, in a configuration where cells touch each other, the ECM shell is already under slight compressive stress, which in reality might not be the case. From the definition of the composite material parameters and the geometrical quantities it is possible to track the position of the cell surface (such as its is possible to calculate in the above point mechanics example the contact point between the two springs). In the extremal case where the cell (including cortex) compression and Young moduli would be much larger than the compression modulus of the ECM, deformation and compression of a cell / ECM unit would basically extend solely on the ECM shell. Approach 3 would be local and is expected to give reasonable approximations in particular if the compressibility of the ECM is much bigger than that of a cell. Performing simulations with a DCM with composite parameters would lead to a different relationship of interaction force versus distance, which then could again be used within CBM simulations.

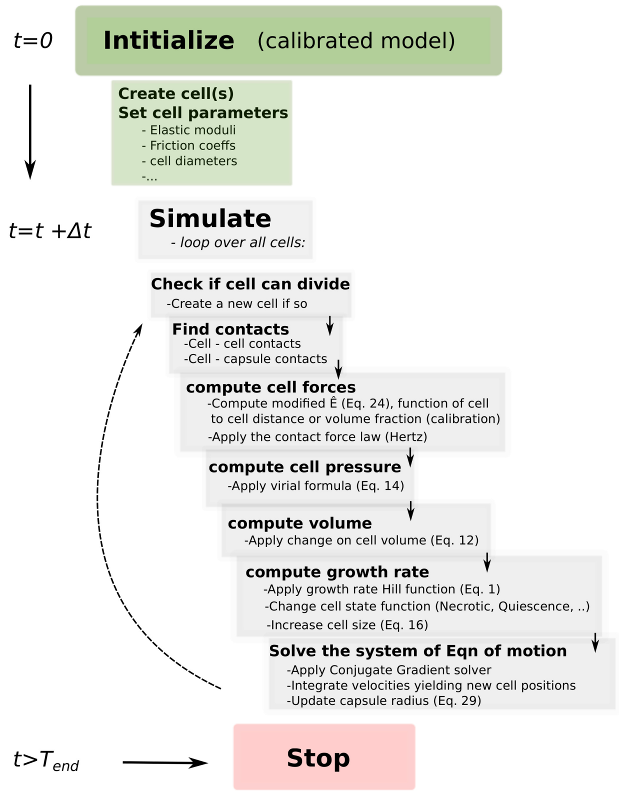

Fig C. Model algorithm flow chart.

Author contributions to this work

DD and PvL conceptualized the modeling strategy, developed the model, and guided the project presented in this paper. PvL and DD supervised JN. PvL, JN and TJ coded the model. PvL and JN performed simulations. KA and PN performed and analysed the experiments. PN concepualized the experiments, PvL and DD wrote the paper, PN gave substantial input for the manuscript. DD and PN raised the funding. The project in this paper originates from a grant project coordinated by PN in Physicancer.

References

1. Drasdo, D and Höhme, S A single-cell-based model of tumor growth in vitro: monolayers and spheroids. Physical Biology. 2005;2:133–147.

2. Delarue M, Montel F, Vignjevic D, Prost J, Joanny JF, Cappello G. Compressive Stress Inhibits Proliferation in Tumor Spheroids through a Volume Limitation. Biophysical Journal. 2014;107(8):1821–1828. doi:10.1016/j.bpj.2014.08.031.

3. Drasdo D, Hoehme S. Modeling the impact of granular embedding media, and pulling versus pushing cells on growing cell clones. New Journal of Physics. 2012;14(5):55025.

4. Smeets B, Odenthal T, Keresztes J, Vanmaercke S, Van Liedekerke P, Tijskens E, Saeys W, Van Oosterwyck H, Ramon H. Modeling contact interactions between triangulated rounded bodies for the discrete element method Computer Methods in Applied Mechanics and Engineering; 2014, 227

5. Alessandri K, Sarangi BR, Gurchenkov VV, Sinha B, Kießling TR, Fetler L, et al. Cellular capsules as a tool for multicellular spheroid production and for investigating the mechanics of tumor progression in vitro. Proceedings of the National Academy of Sciences of the United States of America. 2013;110(37):14843–8. doi:10.1073/pnas.1309482110.

6. Hoehme S, Brulport M, Bauer A, Bedawy E, Schormann W, Hermes M, et al. Prediction and validation of cell alignment along microvessels as order principle to restore tissue architecture in liver regeneration. Proceedings of the National Academy of Sciences. 2010;107(23):10371–10376.

7. Jagiella N, Müller B, Müller M, Vignon-Clementel IE, Drasdo D. Inferring Growth Control Mechanisms in Growing Multi-cellular Spheroids of NSCLC Cells from Spatial-Temporal Image Data. PLoS computational biology. 2016;12(2):e1004412. doi:10.1371/journal.pcbi.1004412.

8. Tinevez JY, Schulze U, Salbreux G, Roensch J, Joanny JF, Paluch E. Role of cortical tension in bleb growth. Proceedings of the National Academy of Sciences of the United States of America. 2009;106(44):18581–6. doi:10.1073/pnas.0903353106.

9. Galle J, Loeffler M, Drasdo D. Modeling the effect of deregulated proliferation and apoptosis on the growth dynamics of epithelial cell populations in vitro. Biophysical journal. 2005;88(1):62–75.

10. Drasdo D Coarse Graining in simulated cell populations. Advances in Complex Systems. 2005;08(02n03):319–363. doi:10.1142/S0219525905000440.

11. Drasdo D, Hoehme S, Block M. On the Role of Physics in the Growth and Pattern Formation of Multi-Cellular Systems: What can we Learn from Individual-Cell Based Models. Journal of Statistical Physics. 2007;128:287–345.

12. Van Liedekerke P, Palm MM, Jagiella N, Drasdo D. Simulating tissue mechanics with agent-based models: concepts, perspectives and some novel results. Computational Particle Mechanics. 2015;2(4):401–444. doi:10.1007/s40571-015-0082-3.

13. Van Liedekerke P, Smeets B, Odenthal T, Tijskens E, Ramon H. Solving microscopic flow problems using Stokes equations in SPH. Computer Physics Communications. 2013;184:1686–1696. doi:http://dx.doi.org/10.1016/j.cpc.2013.02.013.

14. Boal D. Mechanics of the Cell. 2nd ed. Cambridge University Press; 2012.

15. Fedosov DA, Caswell B, Karniadakis GE. Systematic coarse-graining of spectrin-level red blood cell models. Computer Methods in Applied Mechanics and Engineering. 2010;199(29–32):1937–1948. doi:10.1016/j.cma.2010.02.001.

16. Buenemann M, Lenz P. Elastic properties and mechanical stability of chiral and filled viral capsids. Physical Review E. 2008;78(5):051924. doi:10.1103/PhysRevE.78.051924.

17. Galle J, Loeffler M, Drasdo D. Modeling the effect of deregulated proliferation and apoptosis on the growth dynamics of epithelial cell populations in vitro. Biophysical journal. 2005;88(1):62–75.

18. Van Liedekerke P, Tijskens E, Ramon H, Ghysels P, Samaey G, Roose D. Particle-based model to simulate the micromechanics of biological cells. Physical Review E. 2010;81(6, Part 1):61906–61915. doi:10.1103/PhysRevE.81.061906.

19. Beysens DA, Forgacs G, Glazier JA. Cell sorting is analogous to phase ordering in fluids. Proceedings of the National Academy of Sciences of the United States of America. 2000;97:9467–9471.

20. Odenthal T, Smeets B, Van Liedekerke P, Tijskens E, Van Oosterwyck H, Ramon H. Analysis of Initial Cell Spreading Using Mechanistic Contact Formulations for a Deformable Cell Model. PLoS Computational Biology. 2013;9(10):e1003267. doi:10.1371/journal.pcbi.1003267.

21. Basan M, Prost J, Joanny JF, Elgeti J. Dissipative particle dynamics simulations for biological tissues: rheology and competition. Physical biology. 2011;8(2):26014. doi:10.1088/1478-3975/8/2/026014.

22. Schaller G, Meyer-Hermann M. Multicellular tumor spheroid in an off-lattice Voronoi-Delaunay cell model. Phys Rev E. 2005;71(5):51910. doi:10.1103/PhysRevE.71.051910.

23. Ban E, Franklin JM, Nam S, Smith LR, Wang H, Wells RG, Chaudhuri O, Liphardt JT, Shenoy VB. Mechanisms of Plastic Deformation in Collagen Networks Induced by Cellular Forces Biophysical Journal. 2018; 114(2):450-61.
